# Editorial: Post-Acute Sequelae of COVID-19 infection (PASC): Implications for geriatric and neurological care

**DOI:** 10.3389/fnagi.2023.1168720

**Published:** 2023-03-06

**Authors:** Ted Kheng Siang Ng, Vanina Dal Bello-Haas, Ahmed Negm, Elena Philippou, Flávia H. Santos, Patricia C. Heyn

**Affiliations:** ^1^Edson College of Nursing and Health Education, Phoenix, AZ, United States; ^2^School of Rehabilitation Science, McMaster University, Hamilton, ON, Canada; ^3^Cumming School of Medicine, University of Calgary, Calgary, AB, Canada; ^4^Department of Life Sciences, School of Life and Health Sciences, University of Nicosia, Nicosia, Cyprus; ^5^Department of Nutritional Sciences, King's College London, London, United Kingdom; ^6^School of Psychology, UCD Centre for Disability Studies, University College Dublin, Dublin, Ireland; ^7^Center for Optimal Aging, Marymount University, Arlington, VA, United States

**Keywords:** Post-Acute Sequelae of COVID-19 infection (PASC), long COVID, cognitive impairment, rehabilitation, Alzhaimer's disease (AD)

## 1. Introduction

Emerging evidence indicates that many people infected with COVID-19 experience symptoms long after the acute illness phase, characterizing the syndrome Post-Acute Sequelae of SARS-CoV-2 infection (PASC), commonly known as long COVID. Although COVID-19 is primarily a respiratory disease, there are multiple impacts on other systems, including the brain, resulting in cognitive, neuropsychological and neurological impairments. Rehabilitation is thus of paramount importance.

In view of this issue, the guest editorial team thus proposed this *Frontiers in Aging Neuroscience* Research Topic. This Research Topic included nine articles, with six original research and three scoping or systematic review, covering two main areas: (1) Cognitive impairments and (2) Rehabilitation.

## 2. Cognitive impairments

Cognitive impairments are some of the most prevalent PASC symptoms, causing significant disabilities and impacting quality of life.

Chang et al.'s observational study found 72.5% of 40 participants had impairments in at least one cognitive domain examined, defined as ≤ -1.5 standard deviation below measure-specific age- and sex-adjusted norms. The most prevalent impairments were executive function (64.9%), processing speed/attention (52.5%) and working memory (42.5%). COVID-19 related cognitive changes were found in the subacute phase. Limitations include only including patients referred to a psychiatric clinic and the lack of a control group.

Ariza et al. found that compared to the healthy controls (*n* = 109), PASC participants (*n* = 319) scored worse on global cognition, processing speed, language and executive function, learning, and memory tests. The reasonably large sample size represented the full spectrum of COVID-19 severity and excluded participants with comorbidities that could cause cognitive impairment. Limitations include using a less sensitive test to assess visual memory and difficulty finding controls.

Two studies by Guo et al. and Guo et al. analyzed data from the COVID and Cognition Study. In the 2022a paper, to better understand symptom clusters and derive symptom profiles, the characteristics of 181 individuals who had COVID-19 infection were compared to 185 who had not been infected. Principal component analyses (PCA) of 34 initial infection symptoms resulted in a five-factor solution explaining 50.59% of item variance; and PCA of 45 symptoms following initial infection generated a six-factor solution explaining 54.17% of item variance. Initial infection neurological symptoms were found to be significant predictors of self-reported cognitive impairment. As one of the first papers to undertake PASC symptom profiling, strengths included homogeneity of COVID-19 variants with mostly Wild-Type or Alpha-variant SARS-CoV-2. Self-reported symptoms, varied symptoms across time-points, varied response options, lack of vaccination status reporting and laboratory confirmation of infection status, and limited generalizability were limitations.

The 2022b study investigated factors associated with COVID-19 infection that could impact language, executive functions and memory. Cognitive deficits were found to distinguish SARS-CoV-2 patients from non-infected or recovered individuals. Verbal memory deficits and slowness remained even after controlling for demographics and infection severity. Chronic fatigue-like symptoms were predictive of cognitive impairments. Similar limitations to the 2022b study was noted, including relying on online retrospective self-report of symptoms.

Biagianti et al.'s systematic review of neuropsychological assessments for use in people with SARS-CoV-2 found cognitive impairments were prevalent and the likelihood of observing impairments varied depending on the tests used. The MoCA could detect subtle cognitive impairments, while the MMSE could better detect more severe impairments. This finding mirror the finding on higher sensitivity and specificity for detecting mild cognitive impairment using MoCA vs. MMSE (Nasreddine et al., 2005). The 19 studies included had small sample sizes and the tests were unable to identify a specific pattern of impairments related to COVID-19 infection.

## 3. Rehabilitation

The American Congress of Rehabilitation Medicine COVID-19 Task Force summarized health system and rehabilitation recommendations during the COVID-19 pandemic across the care continuum. Negm et al. presented 141 recommendations grouped by (1) setting e.g., rehabilitation inpatient, discharge process, outpatient, (2) health system elements, e.g., rehabilitation equipment/workplace, human resources, telerehabilitation; and (3) precautions for patients and rehabilitation professionals.

Negm et al. described 154 recommendations focusing on acute and post-acute rehabilitation interventions, including geriatric and neurological rehabilitation. Limitations included a lack of empirically-based papers in both the scoping reviews, with available publications comprised of expert opinion or clinical recommendations.

Faieta et al. reported on a 10-item web-based survey that explored the perceptions of 84 caregivers of persons living with dementia. Eighty percent of caregivers reported being isolated from their institutionalized or hospitalized care recipient because of the pandemic, 71.4% were concerned about their care recipient's medical or support care, and 87.2% reported experiencing negative health outcomes. Open-ended comments included mental health concerns i.e., stress, anxiety, depression, inability to sleep, and concerns and worries about their care recipients. Over two-thirds (67.9%) indicated the need for an app to use during COVID-19. The survey was not pilot-tested nor tested for validity; and the use of a web-based platform introduced selection bias and limited generalizability.

Ciro et al.'s study aimed to identify post-hospitalization needs over 30 days using weekly, virtually administered interdisciplinary rehabilitation tools and measures with a convenience sample of 19 people with COVID-19, who hadn't required mechanical ventilation and were discharged from a Level 1 Trauma hospital to home. Initially, participants reported dyspnea were at an increased risk of falls, had difficulties with activities of daily living (ADL) and instrumental activities of daily living (IADL), and had test scores indicative of mild cognitive impairment. At the 30-day follow-up, most participants were independent in mobility and ADLs but had continued IADL needs and cognitive impairments. Limitations include limited generalizability, small sample size, and the missing data.

## 4. Conclusion

Several existing screening and cognitive tests are sensitive to detect cognitive impairments, especially attention, memory and executive function, experienced by people with PASC. Nevertheless, it is unclear to what extent these tests have the specificity to distinguish PASC-related cognitive impairments from other cognitive impairments caused by neurological conditions. Improvements in assessment could facilitate more timely and effective rehabilitation. Since PASC has been shown to be associated with an increased risk of Alzheimer's Disease and Related Dementias (Golzari-Sorkheh et al., 2022), early detection and rehabilitation of PASC-related cognitive impairments are needed to mitigate this emerging public health issue.

## Author contributions

TN: conceptualized and led the writing of this this editorial. PH: conceptualized, recruited associate guest editors, and led the Research Topic. All authors listed were involved in drafting the editorial and made intellectual contribution to the work and approved it for publication.
